# Genomic comparison of multi-drug resistant invasive and colonizing *Acinetobacter baumannii *isolated from diverse human body sites reveals genomic plasticity

**DOI:** 10.1186/1471-2164-12-291

**Published:** 2011-06-04

**Authors:** Jason W Sahl, J Kristie Johnson, Anthony D Harris, Adam M Phillippy, William W Hsiao, Kerri A Thom, David A Rasko

**Affiliations:** 1 Department of Microbiology, Institute for Genome Sciences, 801 W. Baltimore Street, Baltimore, MD, 21201, USA; 2 Department of Pathology, University of Maryland Medical Center N2W69, Baltimore, MD, 21201, USA; 3 Division of Genomic Epidemiology and Clinical Outcomes, Department of Epidemiology and Public Health, 685 W. Baltimore Street MSTF 330, Baltimore, MD, 21201, USA; 4 National Biodefense Analysis and Countermeasures Center, 110 Thomas Johnson Drive, Suite 400, Frederick, MD, 21702, USA; 5 Center for Bioinformatics and Computational Biology, 3125 Biomolecular Sciences Bldg, University of Maryland, College Park, MD, 20742, USA

## Abstract

**Background:**

*Acinetobacter baumannii *has recently emerged as a significant global pathogen, with a surprisingly rapid acquisition of antibiotic resistance and spread within hospitals and health care institutions. This study examines the genomic content of three *A. baumannii *strains isolated from distinct body sites. Isolates from blood, peri-anal, and wound sources were examined in an attempt to identify genetic features that could be correlated to each isolation source.

**Results:**

Pulsed-field gel electrophoresis, multi-locus sequence typing and antibiotic resistance profiles demonstrated genotypic and phenotypic variation. Each isolate was sequenced to high-quality draft status, which allowed for comparative genomic analyses with existing *A. baumannii *genomes. A high resolution, whole genome alignment method detailed the phylogenetic relationships of sequenced *A. baumannii *and found no correlation between phylogeny and body site of isolation. This method identified genomic regions unique to both those isolates found on the surface of the skin or in wounds, termed colonization isolates, and those identified from body fluids, termed invasive isolates; these regions may play a role in the pathogenesis and spread of this important pathogen. A PCR-based screen of 74 *A. baumanii *isolates demonstrated that these unique genes are not exclusive to either phenotype or isolation source; however, a conserved genomic region exclusive to all sequenced *A. baumannii *was identified and verified.

**Conclusions:**

The results of the comparative genome analysis and PCR assay show that *A. baumannii *is a diverse and genomically variable pathogen that appears to have the potential to cause a range of human disease regardless of the isolation source.

## Background

*Acinetobacter baumannii *remains a significant and difficult-to-treat pathogen that causes a range of interactions with the human host from asymptomatic colonization and carriage in the intestinal tract, respiratory tract and skin to invasive infection, such as septicemia, that can result in death. Since the identification of *A. baumannii *as an emerging pathogen, there has been great interest in the rapid development of antimicrobial resistance and nosocomial spread [1,2]. The rapid transmission and subsequent infection of patients with multi-drug resistant (MDR) *A. baumannii *has become a major concern in hospitals and other health care facilities [2]. *A. baumannii *isolates, which routinely cause nosocomial pneumonia, and bacteremia [3-5], have the potential to be resistant to all known antimicrobials [6,7]. The one compounding factor to the rapid spread of antimicrobial resistance is the high transmission rate of *A. baumanni*, especially in the hospital and health care setting [8-10]. These factors have prompted research into the identification of antibiotic resistance markers and subsequent treatment of nosocomial infections (recently reviewed in [11]).

While the examination of *A. baumannii *spread and acquisition of antimicrobial resistance has been the topic of many studies, surprisingly little is known about the pathogenesis of this organism. As would be expected of a mucosal pathogen, biofilm formation has been demonstrated as a virulence factor both for colonization [12] and the resistance of host factors [13]. Russo *et al. *[12] identified a carbohydrate biosynthesis pathway, as well as a protein tyrosine kinase, as virulence factors by using a transposon screen and survival in a rat soft tissue model. This complements the findings by Choi *et al. *that complex carbohydrates were required in the formation of a complex biofilm [14] that is required for full virulence [13]. Few non-carbohydrate features have been conclusively identified as virulence factors. One study demonstrated that the outer membrane protein of *A. baumannii *(AbOmpA) mediates the interaction of epithelial cells and pathogen resulting in persistence of *A. baumannii *in blood [15]. Additionally, mutation of the phospholipase D gene resulted in a decreased ability to invade epithelial cells *in vitro *but did not affect the lung bacterial load *in vivo *[16]. These initial studies have provided limited, but significant insight into the virulence factors of *A. baumannii*. However, further detailed studies will be required to more completely understand the pathogenesis and regulatory networks of *A. baumannii*.

Unlike many other emerging pathogens, *A. baumannii *quickly had genomic sequence generated and now complete genome sequences are available for six *A. baumannii *isolates, with another eight available in draft form (Additional File 1, Table S1). A range of important isolates have been sequenced, including MDR *A. baumannii *isolates, in addition to those that are susceptible to antimicrobials. These datasets have provided the opportunity for comparative studies that examine the MDR phenotype [17-19]. Comparative genomic analyses have focused on large antibiotic resistance islands (RI), such as the 86-kb region found in *A. baumannii *strain AYE (AbaR1) [20], a shorter region found in *A. baumannii *strain ACICU (AbaR2) [18], the AbaR3 region found in *A. baumannii *strain AB0057 [19], the AbaR5 region found in *A. baumannii *strain 3208 [21], and the AbaR6 and AbaR7 regions that consist of deletions compared to AbaR5 [22]. These regions are interesting in that they are chromosomally encoded, encode multiple antibiotic resistance mechanisms and are thought to derive from the integration of plasmids or other mobile elements from other un-related pathogens. Antibiotic susceptible *A. baumanii *isolates can lack these RIs [20,23], suggesting that this region may be important, but perhaps not absolutely required, for the observed antibiotic resistance in every isolate. A recent study has demonstrated that the positive regulation of the AdeL regulator for the AdeFGH resistance-nodulation-cell division (RND) efflux pump contributes to the multidrug resistance phenotype [24]; the AdeABC [25] and AdeIJK [26] efflux systems have also been demonstrated to contribute to a MDR phenotype. These operons are often present in drug susceptible isolates, which demonstrates that is it not the mere gene presence that aids to confer the MDR phenotype, but rather the expression, and in some cases the over-expression, of the efflux pumps that results in the MDR phenotype [24-26]. Further transcriptomic analysis of *A. baumannii *will be required to fully elucidate the mechanisms responsible for the observed resistance phenotype.

Most genomic studies of *A. baumannii *have focused on the genomic composition of invasive isolates [17-20]. The present study includes a comparative analysis of genomes from both invasive and colonization phenotypes to determine if genomic differences could be responsible for the differences in tissue tropism. The *A. baumannii *genome, from antimicrobial resistant or sensitive isolates, contain a relatively large number of mobile elements suggesting there are mechanisms for rapid integration of foreign DNA that may expand the host range and/or virulence of this pathogen. In this study, draft genome sequences were obtained from isolates of *A. baumannii *from human blood, peri-anal, and wound samples. The isolates were demonstrated to be different with multilocus sequence typing (MLST) and pulsed-field gel electrophoresis (PFGE) methods. Further analysis with comparative whole genome sequencing and comparative genomics approaches were employed to determine how these isolates are related in a phylogenetic context, and to identify genomic regions that may aid in establishing colonization and/or invasive infection.

## Methods

### Cohort Description

The University of Maryland Medical Center (UMMC) is a 656-bed tertiary-care hospital located in Baltimore, Maryland, with a 19 bed surgical intensive care unit (SICU) and a 29 bed medical intensive care unit (MICU) that provides care to adult patients who have acute or potentially life-threatening medical conditions. This study utilized an existing and ongoing prospective cohort of adult patients admitted to the SICU and MICU at the UMMC from January 2008 to June 2008 who had routine (admission, weekly and discharge) peri-anal surveillance cultures obtained [27]. Bloodstream isolates were obtained from cohort participants with an imipenem-resistant *A. baumannii *blood stream infection (BSI). Peri-anal samples for culture were obtained using Staplex II cotton swabs (Staplex, Etobiocoke, Ontario, Canada), which are used to swab the peri-anal area in a circular motion moving from the rectum out [27]. Surveillance swab cultures were stored by placing swabs in tryptic soy broth containing 30% glycerol and stored at -80°C to allow for future recovery of isolates [28].

### *A. baumannii *isolation

Clinical isolates (blood and wound) were isolated from UMMC's microbiology laboratory using standard procedures. Peri-anal swabs were cultured for *A. baumannii *in the research laboratory by streaking swabs onto MacConkey agar (Remel, Lenexa, KS) supplemented with 6 µg/ml of imipenem and incubated at 37°C for 24 to 48 hours. Recovery of organisms from archived frozen peri-anal specimens has been shown to be 98% effective [28]. Non-lactose fermenting organisms (white colonies on MacConkey agar) were further identified as *A. baumannii *using API NE 20 Identification Strips (BioMérieux Inc.; Hazelwood, MO, USA) or Vitek II (BioMerieux, Durham, NC).

### Strain selection

We selected one isolate from blood, peri-anal swab and a wound for further characterization. These three isolates selected were all isolated during a time period in 2008 where a localized outbreak of *A. baumannii *was ongoing at the UMMC. UMB001 was a virulent outbreak strain isolated from the blood of a patient in the SICU; UMB002 was a strain isolated from a peri-anal sample that was not part of the outbreak but was isolated at the same time in the MICU; UMB003 was also present at the time of the outbreak, but was isolated from a wound sample and was not related to the outbreak strain. In addition to the isolates selected for genome sequencing, a collection of 74 *A. baumannii *isolates was used for the polymerase chain reaction (PCR) screening of genomic markers specific to either invasive or colonization isolates (see below). This collection included 20 randomly sampled peri-anal surveillance isolates collected as part of an ongoing cohort of intensive care patients; 18 isolates were collected from February to May 2008 and 2 isolates were historical isolates (2006 and 2007) from patients later known to have *A. baumannii *bloodstream infections in 2008. The collection also included 54 invasive isolates from blood infections at UMMC collected from 9/2008-6/2010.

### Antibiotic Resistance Methods

Antimicrobial susceptibility testing was performed on all isolates by the Kirby-Bauer disk diffusion method. Susceptibility testing was performed and interpreted in accordance with Clinical and Laboratory Standard and Institute (CLSI) guidelines [29].

### Pulsed-field gel electrophoresis (PFGE)

PFGE was performed on the three selected isolates. PFGE was performed following the protocol described at http://www.cdc.gov/pulsenet/protocols.htm with slight modifications described below [30]. Agarose plugs were prepared as previously described by Maslow et al [31] and the DNA was digested with *ApaI *[32]. DNA was subsequently separated in 1% agarose on a contour-clamped homogeneous-field machine (CHEF-DR II, Bio-Rad, Richmond, CA). Electrophoresis was performed at 6 V/cm for 18.5 hours, with pulse times ranging from 7-20 seconds. Following electrophoresis, the gels were stained with ethidium bromide and photographed under ultraviolet illumination. Interpretation of PFGE was preformed using the criteria outlined by Tenover et al. [33], where band patterns are visually compared and classified as indistinguishable (clonal), closely related (clonal variants, three or less band differences), possibly related (four to six band differences) and unrelated (seven or more band differences).

### DNA extraction, genome sequencing, and annotation

Genomic DNA was extracted from the three cultures grown overnight at 37ºC using standard methods [34]. Genomes were sequenced with the 454 Titanium platform and raw reads were assembled with Newbler (454 Life Sciences). The resulting assembly was annotated with an automated annotation pipeline at the Institute for Genome Sciences (http://www.igs.umaryland.edu/). Assembly details are included in Additional File 2, Table S2.

### Multiple locus sequence typing (MLST)

The gene sequences for seven housekeeping genes (*gltA, pyrG, rplB, recA, cpn60, fusA, rpoB*) were informatically extracted from genomic contigs with Mugsy [35] and were compared to allele profiles in the *A. baumannii *PF8MLST database (http://www.pasteur.fr/recherche/genopole/PF8/mlst/Abaumannii.html); sequence types were then assigned for matching profiles.

### Blast score ratio (BSR) analysis

A BSR analysis [36] was performed on the predicted proteome of the three genomes included in this study and 6 finished *A. baumannii *genomes (Table 1). Peptides were classified as unique for a BSR > 0.80, divergent for a BSR < 0.80 and > 0.40, and unique for a BSR < 0.40 as described in previous multi-genome analyses [37-39].

**Table 1:** MLST and genic information for each sequenced isolate

Isolate	Isolation site	PR8MLST ST^a^	number of genes	GC %
UMB001	blood	2	3858^b^	38.87
UMB002	perirectal	16	3831^b^	39.10
UMB003	wound	25	4072^b^	39.05
AB0057	blood	1	3912	39
AB307-0294	blood	1	3542	39
ACICU	cerebrospinal fluid	2	3749	39
ATCC_17978	meningitis	77	3453	38
AYE	urine	1	3760	39
SDF	body louse	17	3598	39

^a^http://www.pasteur.fr/recherche/genopole/PF8/mlst/Abaumannii.html

^b^potentially includes plasmid sequence

### Resistance island visualization

The AbaR1 gene sequence was downloaded from Genbank (Locus ID CT025832). Each genome was aligned against this sequence with NUCmer [40]. The resulting percent identity plot was generated showing conservation over the entire length of the gene, as has been done recently [41].

### Whole genome alignment and phylogenetics

A whole genome alignment was performed with Mugsy [35] on a total of 23 available *A. baumannii *genomes (Additional File 2, Table S2) using a maximum gap length between orthologous blocks of 100 base pairs. Columns with gaps were removed from the alignment with mothur [42] to generate an ungapped multiple sequence alignment. For phylogenetic studies, extracting polymorphic positions reduced the size of the alignment. A phylogenetic tree was then inferred on this reduced alignment with FastTree [43], with 1000 boostrap replicates and using *Acinetobacter calcoaceticus *as the outgroup.

### Polymerase chain reaction (PCR) screening

The collection of 74 *A. baumannii *isolates was screened by PCR for the genomic regions identified from the Mugsy alignment as being associated with the colonization or invasion phenotypes. Single colonies were grown overnight in Luria broth at 37ºC. Aliquots of the overnight cultures were boiled at 94ºC for 10 minutes, and then the cellular debris was removed by centrifugation for 5 min at 4000 × RPM; this crude cell lysate was then used as the PCR template. PCR primers were designed with Primer3 [44]. A list of primers used in this study is shown in Table 2. Reaction conditions were as follows: 95ºC for 5 minutes followed by 30 cycles of 94ºC for 30 s, 58ºC for 30 s, and 72ºC for 45 s.

**Table 2:** Oligos and genomic targets used in this study

Primer name	Forward (5'-3')	Reverse (5'-3')	gene target	gene target accession	Phenotype target
AB_UNI	CTTTCAGGKAGTATTGGTCT	CTCATTAGAGTTCACCGAAG	hypothethical protein	ACJ58112	all
AB_HYP	CGTCGGTCGGATCCGTGTAT	AAGTAAAGTGGCAGGCGCTT	hypothetical protein	ACJ57861	invasion
AB_INV	ATCCATTAGACCTCCCAATC	CATTGTTGATGTGAACTGGA	hypothetical protein	ACJ42779	invasion
AB_HEM	TCTCATCTAAAGCACCACCC	TAACCAGTGCTGGTGACATC	hemolysin	ZP_04662166	colonization
AB_COL	TCCAATGCCAAATCTTTACT	TGTGTTGAATAACCCTCAAA	alkyl sulfatase	ZP_04663686	colonization
AB_ALK	TTCCTGTTATAAGATTGGGAGAA	TTCTGATCCGAGTGATTTATTG	alkyl sulfatase	ZP_04663686	colonization
AB_INT	TATCCTTCAGCTTACCCTTCTT	TTTCGACATGTGGTAGACTGTT	Integrase	ZP_04661979	colonization
pOX	TGGCGATCAATTCTTACTTT	CGTAACGAAACGAAGTAATG	phenol hydroxylase	ZP_05824220	none

### Accession numbers

Genome sequence data are available in GenBank under accession numbers AEPK00000000 (UMB001), AEPL00000000 (UMB002), and AEPM00000000 (UMB003).

## Results

### *A. baumannii *isolate selection

Three isolates were chosen for sequencing: a colonizing isolate from the peri-anal site (UMB002), an outbreak isolate from the bloodstream (UMB001), and a non-outbreak wound isolate (UMB003). Each of the isolates has a different PFGE pattern (Additional File 3, Figure S1), providing evidence that, while these isolates were obtained from the same hospital setting in a limited timeframe, they do not represent highly similar clones that are part of a single outbreak.

### MLST analysis

The allelic profiles were obtained from the genome sequencing projects of the three new genomes analyzed in this study. The PF8MLST (http://www.pasteur.fr/recherche/genopole/PF8/mlst/Abaumannii.html) system assigned the genomes into sequence types (STs) 2 (UMB001), 16 (UMB002), and 25 (UMB003) (Table 1). The comparison of these sequence types to the previously sequenced genomes identified that there is not a consistent MLST pattern associated with the isolates from invasive or colonization sources (i.e. there is not a predominant sequence type for invasion isolates). Additionally, the MLST sequence type did not extend to other phenotypes, for example, strain AB0057 and AB307-0294 are both ST1; AB0057 shows a MDR phenotype, while AB307-0294 shows a susceptible phenotype.

### Whole genome phylogenetic analysis

**Figure 1:**
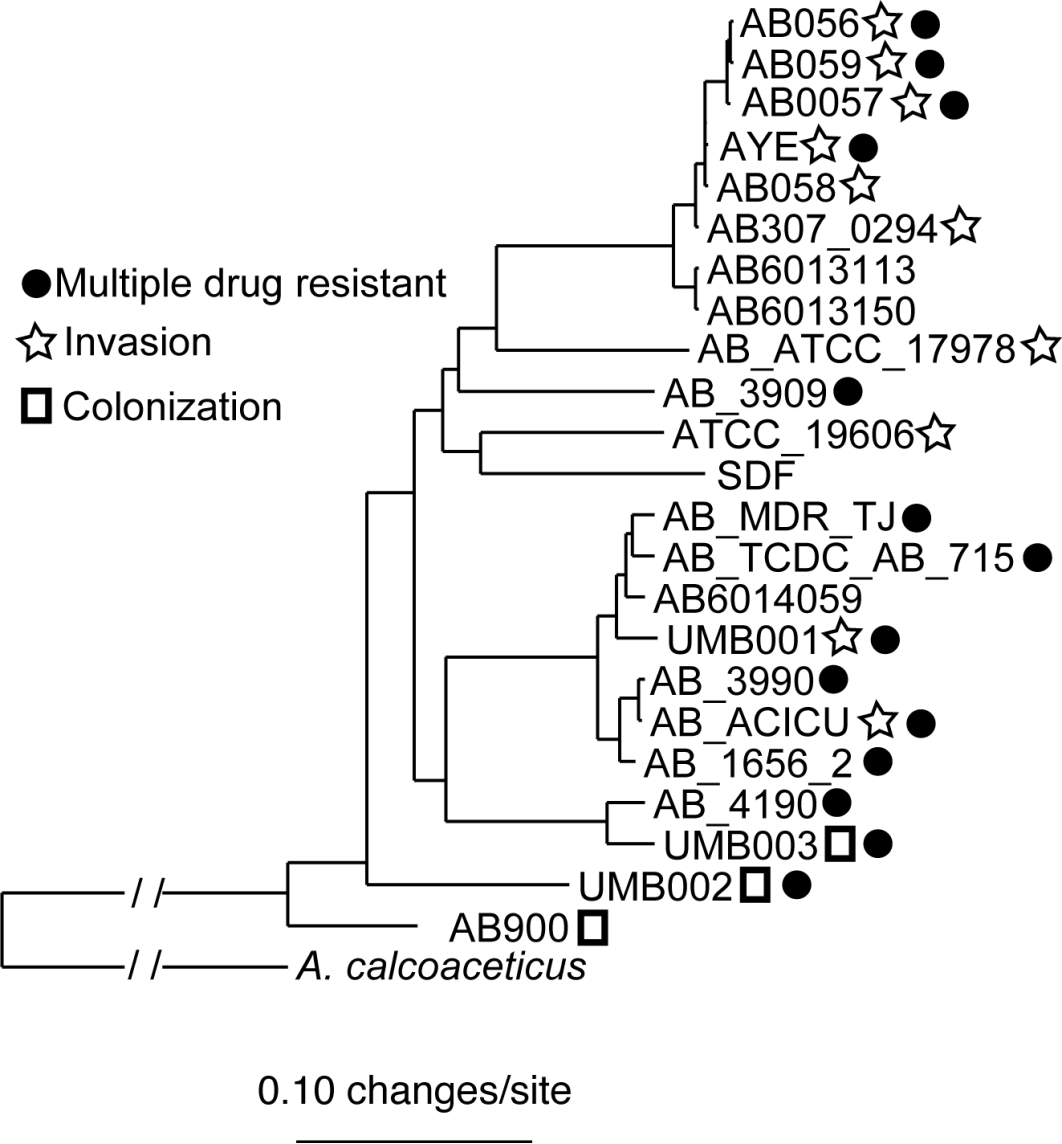
**A phylogenetic tree of all sequenced *A. baumannii *genomes constructed using whole genome alignments**. Open boxes represent those strains from the colonization phenotype (wound, skin) and the star represents those isolates from the invasive phenotype (blood, urine); the black dots indicate the MDR strains. For genomes with no phenotype description, the phenotype is either unknown or does not affect humans. The tree is constructed based on the >300,000 variable locations in the genome alignment. The support values at all nodes are > 90%. There is little clustering based on the isolation source or MDR status based of the isolates suggesting that these traits may be being acquired via horizontal gene transfer and are not highly conserved.

In an effort to assess the phylogeny of these genomes in a gene/annotation independent manner we used the whole genome comparative analysis described previously [34]. The whole genome alignment method identified 2.25 Mb of homologous sequence data shared between all available *A. baumannii *isolates (Additional File 1, Table S1). From this multiple sequence alignment, we identified 330,631 polymorphic positions (14.7% of the total alignment). As has been demonstrated previously [19], multi-drug resistant (MDR) strains do not share a monophyletic origin (Figure 1). Limited genetic variation was observed between all *A. baumannii *genomes; an uncorrected distance matrix for this alignment showed < 2% nucleotide variation for all 17 genomes.

### *A. baumannii *pan-genome analysis

**Figure 2:**
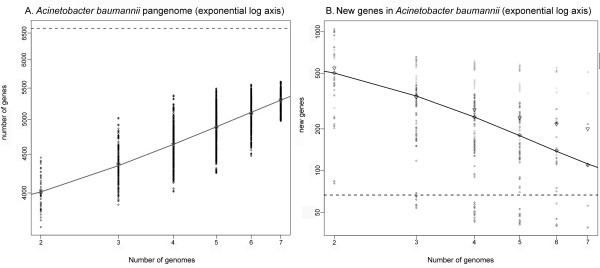
**Pan-genome analysis of the complete genomes of *A. baumannii***. Panel A demonstrates the pan genome size of *A. baumannii *as calculated based on the power regression model. The plot shows an open pan-genome that predicts the total genome space to contain ~ 6500 genes. Panel B shows the rate of identification of new genes in each genome is approximately 66 genes per new genome at extinction. These values are based only on the complete genomes as it is difficult to predict complete genome size from incomplete genome data.

Using a pangenome analysis pipeline maintained at the Institute for Genome Sciences [45-47], we estimated the size of the *A. baumannii *pangenome based on 6 complete, publicly available *A. baumannii *genomes (Table 1). We estimate the pangenome size to be approximately 6500 genes (Figure 2A) and approximately 66 new genes are expected with the sequencing of each additional genome (Figure 2B). These values are similar to other *A. baumannii *pan-genome calculations [19].

### Blast score ratio (BSR) analysis

The BLAST score ratio analysis will identify the level of conservation of genes that have been predicted in any set of genomes [36]. Using a conservative BSR threshold of 0.4 (similar to <30% amino acid identity over <30% of the peptide) we identified the unique genes in each of the new genomes sequenced in this study when compared to the complete genomes in GenBank (Additional File 2, Table S2). The BSR analysis for the genomes sequenced in this study identified 180 unique genes in UMB001, 290 in UMB002 and 496 in UMB003. These genes and functional annotations for UMB001-UMB003 are included in Additional Files 4, 5, 6, 7, Tables S3-S6), respectively. For each of these genomes there is no functional annotation for ~ 60% of the unique gene set (UMB001, 106 genes = 58.9%; UMB002, 182 genes = 62.8% UMB003, 288 genes = 58.1%). The majority of genes with functional annotation are similar to genes that are involved in horizontal gene transfer; these genes include transposases or genes involved in Type IV secretion systems that constitute plasmid mobility and conjugation systems. These plasmid transfer genes further suggest the presence of plasmids in these isolates.

The largest unique gene set was observed in UMB003. Interestingly these genes with functional annotation have similarities that suggest recent lateral gene transfer between not just other *Acinetobacter *species, but other unrelated species. A chloramphenicol acetyltransferase gene was identified that showed high homology (99%) to a *Klebsiella pneumoniae *homolog [48]. While this gene has been identified in another *A. baumannii *isolate by single gene sequencing [49], the previous version demonstrates a much lower level of homology (81%); this gene is not present in any publically available *A. baumannii *complete or draft genome sequence. Interestingly, in UMB003 a phenol hydroxylase protein complex was identified. This complex is responsible for the breakdown of phenol, the base for many disinfectants; a homologous gene cluster (99% identity) was originally identified in an *Acinetobacter calcoaceticus *genome isolated from oil refinery wastewater [50]. A primer set was designed for a conserved protein in the phenol hydroxylase complex. A screen of the 74 *A. bumannii *isolates showed that 25 of them were positive for presence of the amplified gene fragment. A BLAST search of this conserved fragment against all 23 sequenced *A. baumannii *genomes only showed hits against UMB003 and the draft genome sequence AB_4190 [51].

### Antibiotic resistance mechanisms

Clinical laboratory testing identified resistance in all three isolates to several antibiotics including amikacin, ceftazimide, and gentamicin (Table 3). A genomic comparison identified that each isolate contained a gentamicin 3'-acetyltransferase and an RND type efflux pump (AdeT) previously demonstrated to be involved in aminoglycoside resistance [52]. All Isolates contained an AmpC cephalosporinase, which would help explain the clinical observation of ceftazidime resistance [53]; the insertion sequence ISAbar1, which has been associated with over-production of AmpC [54], was also present in UMB001 and UMB002. The oxacillinase like-gene *bla *_OXA-69_, which has the ability to hydrolyze carbapenem [55], was present in all genomes.

**Table 3:** Antibiotic resistance in sequenced isolates

	Isolate			
		
Antibiotic	UMB001	UMB002	UMB003	Potential mechanism(s)
Amikacin	R	R	R	Efflux
Gentamicin	R	R	R	Efflux
Ampicillin Sulbactam	R	S	R	Oxa69, AmpC + IS element, efflux
Ceftazidime	R	R	R	Oxa69, AmpC + IS element, efflux
Cefepime	R	R	R	Oxa69, efflux
Piperacilin Tazobactam	R	R	R	Oxa69, AmpC + IS element, efflux
Imipenem	R	R	I	Efflux
Ciprofloxacin	R	R	R	Efflux
Tigecycline	I	I	I	AdeABC
Sulfamethoxazole trimethoprim	R	R	R	dhfrI/dhfrII - dihydrofolate reductase

R = resistant

S = susceptible

I = intermediate

### Resistance islands (RIs)

**Figure 3:**
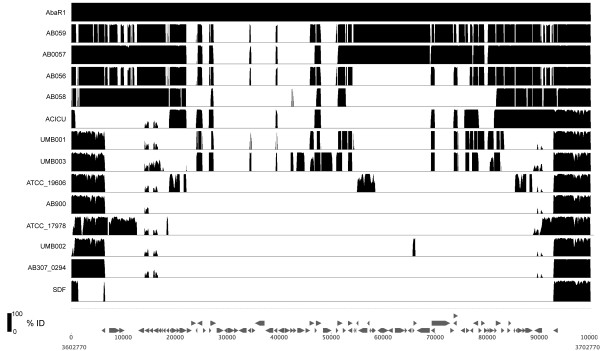
**A percent identity plot of all genomes compared to the resistance island AbaR1 in strain AYE**. The height of each peak represents percent identity (755-100%). The annotation for the resistance island is shown at below the percent identity plot. The genomic coordinates at the bottom of the figure indicate the position of AbaR1 in the AYE chromosome.

Resistance islands are common in *A. baumannii *as genomic locations for the acquisition and accumulation of genes required for antibiotic resistance [20,22]. The most well studied of these RIs is AbaR1 [20]. We utilize the AbaR1 structure as a reference as it appears to be the largest of the RI and all others appear smaller, however it must be noted that the complete AbaR1 structure has not been observed in any other isolate to date. Comparison of all *A. baumannii *genomes in this genomic region identifies a wide spectrum of genomic conservation (Figure 3). Limited, but highly conserved regions (BSR > 0.80) of the AbaR1 RI are present in UMB001 and UMB003, but absent in UMB002 (Figure 3). Bioinformatic analysis identified that UMB001 and UMB003 contain an aminoglycoside resistance protein and the transposase TN21 that are present in the prototype AbaR1. UMB002 contains only 5 genes from AbaR1, and none of them associated with antibiotic resistance. An analysis of genomic contigs that contain *comM *from all three UMB strains failed to identify additional genes from known RIs; this suggests that homologous genes to those in known RIs are either in previously unknown RIs, or are not present in a genomic island.

### Efflux pumps

Previous studies have demonstrated the importance of efflux pumps at removing antimicrobials from the cell and conferring the MDR phenotype. These pump systems are present in the three genomes sequenced in this study, but are also present in genomes of drug susceptible isolates. Three systems have been demonstrated to contribute to the MDR phenotype, AdeABC, AdeFGH and AdeIJK [24-26]. The three genomes sequenced in this study contain the *adeFGH *operon, as well as the gene encoding the regulator, AdeL. A multiple sequence alignment of the predicted *adeL *coding sequence demonstrated no single nucleotide polymorphisms (SNPs) that were unique to either the MDR or drug susceptible phenotypes (data not shown). Additionally, each of the three new genomes contained highly conserved *adeA *and *adeB *genes from the AdeABC efflux pump complex; however, UMB002 and UMB003 lack the gene encoding the outer membrane protein AdeC [56]. A multiple alignment of the AdeABC regulator, AdeR [57], also showed no MDR-specific SNPs.

### Identification of conserved genomic fragments

Detailed comparative genomic analysis identified one gene encoding a hypothetical protein (accession YP_001713249) that is conserved in all 23 *A. baumannii *genomes and has no identified homologue in 12 other *Acinetobacter *species genomes. A pairwise alignment demonstrates that there is a high level of conservation for this peptide (Additional File 8 Figure S2). PCR primers were designed from this coding region (Table 2) and a PCR based screen of 74 putative *A. baumannii *isolates identified 73 positive amplicons (98.6% sensitivity). A BLAST homology search identified the closest homolog as a protein of unknown function with 47% peptide identity in the actinomycete *Catenulispora acidiphila*.

### Colonization and invasion genotype analysis

The majority of *A. baumannii *genomes sequenced to date demonstrate an invasion phenotype, as they are isolated from fluids within the human body (Table 1 and Additional File 1, Table S1). In fact, only one previously available draft genome, AB900, was isolated from what the present study defines as a colonization isolate. In the case of AB090, the colonization site was the perinea [19]. The current study adds two draft genomes (UMB002, UMB003) that were isolated from colonization of the peri-anal region and a wound, respectively.

**Figure 4:**
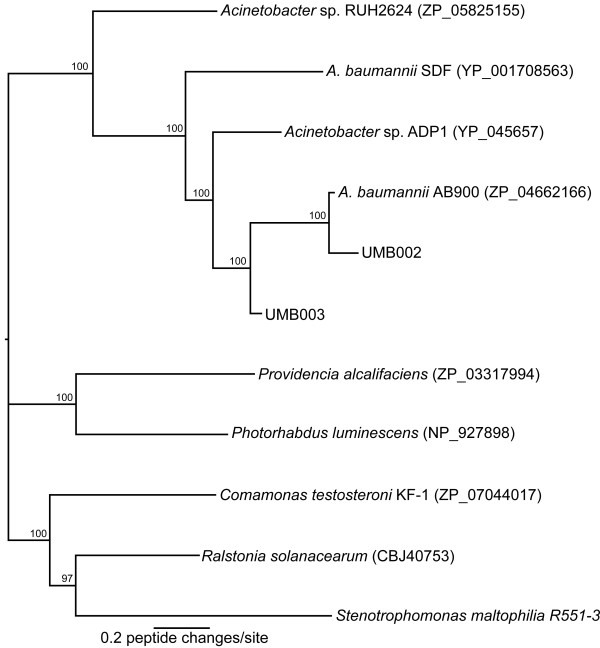
**A phylogenetic tree of hemolysin proteins**. Hemolysin proteins were obtained from GenBank and aligned. The phylogenetic tree was inferred with FastTree 2, with 1000 bootstrap replicates. Numbers at nodes indicate bootstrap support values. Numbers next to isolates are Genbank accession numbers. The diagram indicates that the hemolysin gene in *A. baumannii *has not been acquired by horizontal gene transfer, but rather it appears to be ancestral the *Acinetobacter *species.

A comparative whole genome approach was employed to determine if the organisms with either a colonization (AB900, UMB002, UMB003) or invasion phenotype (AYE, AB0057, AB307-0294, ACICU, ATCC 17978, UMB001, AB056, AB058, AB059) contain unique genomic content. Three additional *A. baumannii *genomes (AB6013113, AB6013150, AB6014059) were not included in this analysis due to their uncertain isolation location. The results of the whole genome alignment approach demonstrate that unique genomic regions in the invasion isolates consisted mainly of hypothetical proteins (Table 4). There were also identified regions within the colonization isolates that were found to be absent in the invasion isolates. One region in the colonization genotyped isolates contained a large (>5000 aa) putative hemaglutinin/hemolysin-related protein related to a gene that was also present in the body louse strain *A. baumanii *SDF [20]. Phylogenetic analysis of homologous genes in other pathogens showed that this gene has not been horizontally transferred recently into the *Acinetobacter *species group (Figure 4). We developed a PCR-based assay to determine if these genetic features were consistently present in a larger group of colonization or invasive isolates. A screen of 74 *A. baumannii *isolates with primers specific to genes present in either colonization or invasion isolates (Table 2) demonstrated that the genes common to the isolates from each phenotype in the genomic analysis were not restricted to that phenotype in a broader population based analysis (Table 4). Only the two sequenced colonization isolates amplified with the "colonization specific" primer sets. Furthermore, the primers designed from "invasion specific" genomic regions showed no specificity to *A. baumannii *obtained from blood compared to peri-anal isolates.

**Table 4:** PCR screening results for genomic markers in 77 isolates

Primer_set	phenotype target	positive invasion^a^	positive colonization^b^	no amplification
AB_UNI	all	55 (100)	21 (95)	1 (1)
AB_HYP	invasion	51 (94)	19 (86)	6 (8)
AB_INV	invasion	53 (96)	20 (91)	2 (3)
AB_HEM	colonization	0 (0)	2 (3)	75 (97)
AB_COL	colonization	0 (0)	2 (3)	75 (97)
AB_ALK	Colonization	0 (0)	2 (3)	75 (97)
AB_INT	Colonization	0 (0)	2 (3)	75 (97)

^a^N = 55

^b^N = 22

## Discussion

The results of this study, which uses whole-genome comparative methods, demonstrate that the *A. baumannii *genome has the potential to accommodate a range of pathogenic phenotypes. This is the first study to use whole genome alignment and phylogenetic methods to classify *A. baumannii*. While methods such as MLST and PFGE can identify differences between related isolates, whole genome sequencing allows us unprecedented insight into the evolution and relatedness of cultured isolates. The variable nature of the *A. baumannii *pangenome [7,19,20,58] suggests that additional whole genome sequence analysis will help to identify additional genes associated with antibiotic resistance and pathogenesis.

Our results demonstrated a wide variety of sequence types circulating within an individual hospital. With the PF8MLST typing system, UMB001 was assigned to ST2, which also contains the outbreak, multi-drug resistant isolate ACICU, and is one of the most commonly encountered STs. UMB003 was assigned to ST25 that contains outbreak strains isolated in Greece and Turkey [59]. The UMB002 sequence type, ST16, is a much rarer sequence type; for example, out of a study of ~130 *A. baumanniii *isolates, only one, an outbreak isolate from the Netherlands, has the same allele profile [60]. Each of these *A. baumannii *isolates was circulating within UMMC during the same limited time window, providing the potential for interaction and additional isolate-to-isolate horizontal gene transfer.

From a clinical perspective, the utility of typing a new isolate is uncertain and provides little, if any data that is utilized in patient treatment; however, genomics can provide insights that may impact clinical decisions. For example, a recent study examined at micro-evolution between three (AB056, AB058, AB059) phylogenetically similar clinical isolates [58], but found significant divergent susceptibilities to antimicrobials. This is highlighted in the current study in that included on the same phylogenetic branch and showing very little evolutionary distance are isolates expressing both MDR and susceptible phenotypes (Figure 1). This supports the mounting evidence that whole genome sequencing is necessary as a diagnostic tool, because it provides more detailed information than MLST or other typing methods, and it provides additional information about the genetic machinery necessary for antimicrobial resistance and pathogenesis. A recent study by Lewis *et al. *[61] utilized whole genome sequencing of *A. baumannii *for the investigation of a hospital outbreak in UK. This is one of the first uses of whole genome sequencing in an outbreak investigation or epidemiological setting. The fine scale detail analysis allowed by whole genome sequencing extended the findings of traditional epidemiological studies that were underway. The combination of traditional methodologies with next generation sequencing technologies will provide detailed analyses of these pathogens.

*A. baumannii *isolates have been identified from a wide range of human clinical sources including urine, blood, cerebrospinal fluid, and wounds [19,20,58]. One of the goals of this work was to identify if there were unique genetic features that could be attributed to a specific pathogenic phenotype. Bioinformatic analysis of available genomes tested the hypothesis that the gene repertoire of an invasion isolate (blood, urine) would be different than a colonization isolate (peri-anal, fecal matter, wound). In this study, peri-anal isolates were considered to be a colonization phenotype; surveillance from the peri-anal site has been shown in prior studies to represent the gastrointestinal flora (90% sensitivity, 100% specificity compared to stool samples) [62]. Furthermore, studies have shown that bacteria found on peri-anal surveillance culturing often persist for prolonged periods [63,64] and are associated with subsequent invasive disease [65].

Genetic features were identified that were exclusively present in genomes from each phenotype, which suggested that these genes encode proteins that aid in either *A. baumannii *invasion causing clinical infection or colonization. For example, a putative hemolysin gene was present in all colonization phenotype isolates (Figure 4), but was absent in all invasion phenotype isolates. A phylogenetic analysis demonstrated that the gene is present in ancestral *Acinetobacter *lineages and does not appear to be a recent introduction via horizontal transfer (Figure 4). However, when genes unique to each isolation phenotype were screened against a broader collection of isolates including colonization and invasion isolates, no correlation between phenotype and the pattern of gene presence/absence was observed. The design of the PCR-based assays was based on a limited number of isolates from each phenotype, and it's not surprising that the presence/absence pattern fails in a broader analysis. These findings demonstrate that results based solely on comparative genomic analysis of a limited number of genomes may not be broadly applicable to diverse culture collections, especially an organism with the genomic mosaicism of *A. baumannii*. The study highlights the difficulty in correlating clinical phenotypes with genotypes in some pathogens.

While the comparative analysis did not identify a consistent genotype associated with each of the isolation source groups, this study did identify a number of very interesting traits that highlight the genetic variability of *A. baumannii *and could potentially expand the survival of this pathogen in the hospital setting. For example, genes associated with phenol metabolism were exclusively found in isolate UMB002; the absence of these genes in other *A. baumannii *isolates suggests that these genes were acquired through horizontal gene transfer as they are similar to genes found in another *Acinetobacter *species isolated from oil refinery wastewater [50]. However, it is possible that this isolate was selected for over time, as the primary surface cleanser at UMB contains nonoxynol, which is phenolic based, and an organism that would potentially be resistant to these types of cleaners would have an increased rate of transmission; this is supported by the fact that this complex is more frequently found in UMMC isolates, compared to the examined genomic space of the *A. baumannii *species. Additional experimental work is needed to verify that UMB002 can metabolize phenolic compounds, but the presence of these genes suggests a mechanism by which this isolate may persist and thrive on hospital surfaces.

In contrast to the variable genomic features highlighted here, we also identified a gene that encodes a large, conserved peptide that was found in all sequenced *A. baumannii *genomes; no homologous peptide was found in any other sequenced *Acinetobacter *species. A PCR screen demonstrated that this gene is present in *A. baumannii isolates *(~99% of isolates screened), suggesting that it can be used as a biomarker for the positive identification of clinical *A. baumannii *isolates. Additional population screening will help determine how well conserved this peptide is among a more diverse collection of clinical isolates.

## Conclusions

Using whole genome phylogeny we have refined the genetic relationships of the previously sequenced *A. baumannii *isolates and new isolates examined in this study. These findings identified that much of the *A. baumannii *variability can be attributed to horizontal gene transfer, but it cannot account for all of the observed variability. In all sequenced genomes, we identified a conserved genomic region unique to *A. baumannii *that could serve as a biomarker for new clinical strains. In addition, we identified unique regions in each of the sequenced genomes, as well as regions unique to both colonization and invasive phenotypes. These regions were examined by PCR in a larger population size and shown not to be consistent with the isolation source. These results further highlight the genetic variability of *A. baumannii *and emphasize that the genetic potential of this pathogen is vast enough to infect and/or colonize multiple human body sites.

## Authors' contributions

JWS preformed novel bioinformatic analyses, designed experimental procedures, performed molecular testing and wrote the manuscript. JKJ, KAT and ADH preformed the clinical study and collected the isolates that are included in this study, characterized the isolates and aided in the writing of the manuscript. AMP and WWH preformed novel bioinformatic analyses integral to the manuscript. DAR designed experimental procedures, performed novel bioinformatic analyses and helped write the manuscript. All authors have read and approved the final manuscript.

## Supplementary Material

Additional file 1 ***A. baumannii *genomes and isolation sources**. A description of published genomes used for comparative genomics in the present study Click here for file

Additional file 2 **Genome Assembly Statistics**. Assembly statistics for the 3 genomes sequenced in this study Click here for file

Additional file 3 **Pulsed field gel electrophoresis (PFGE) profiles**. A picture that shows PFGE profiles of the three strains sequenced in this study Click here for file

Additional file 4 **BLAST score ratio analysis**. A table of unique, divergent, and unique gene numbers as identified by blast score ratio (BSR) analysis Click here for file

Additional file 5 **UMB001 unique genes**. A table of unique genes identified in UMB001, based on a BSR value of less than 0.4 Click here for file

Additional file 6 **UMB002 unique genes**. A table of unique genes identified in UMB002, based on a BSR value of less than 0.4 Click here for file

Additional file 7 **UMB003 unique genes**. A table of unique genes identified in UMB003, based on a BSR value of less than 0.4 Click here for file

Additional file 8 **Protein alignment of a conserved, *A. baumannii*-specific, hypothetical protein**. Multiple sequence alignment of a conserved peptide identified in this study for all sequenced *A. baumannii *isolates Click here for file
