# Supplementary figures and images for: Genomic comparison of multi-drug resistant invasive and colonizing Acinetobacter baumannii isolated from diverse human body sites reveals genomic plasticity
Source: BMC Genomics. 2011 Jun 4;12:291. doi: 10.1186/1471-2164-12-291 (PMC3126785; doi:10.1186/1471-2164-12-291)

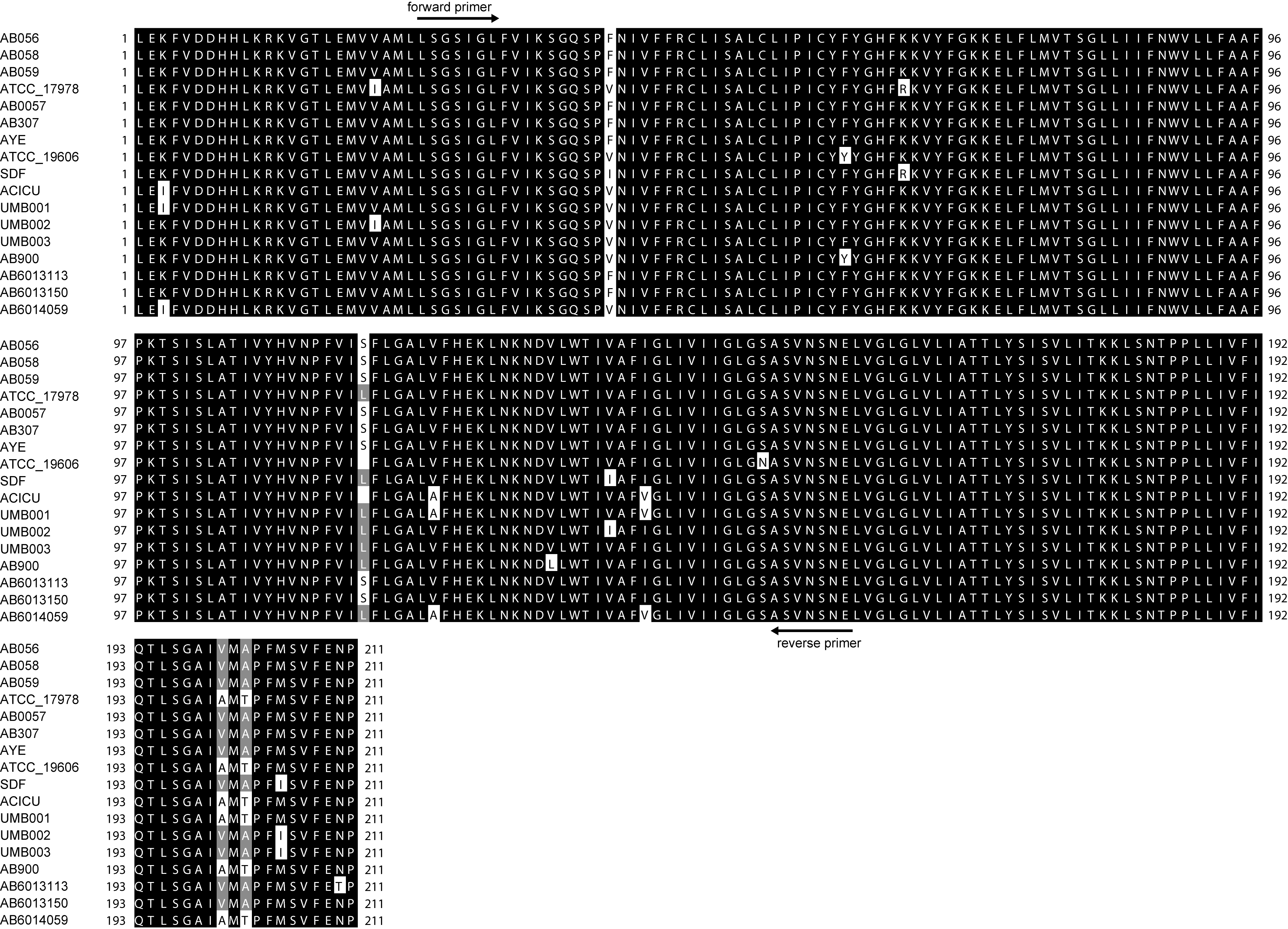

Supplement: Additional file 8 — Protein alignment of a conserved, A. baumannii-specific, hypothetical protein. Multiple sequence alignment of a conserved peptide identified in this study for all sequenced A. baumannii isolates [file 1471-2164-12-291-S8.TIFF]
